# Ultrahighly Sensitive Surface-Enhanced Raman Spectroscopy Film of Silver Nanoparticles Dispersed in Three Dimensions on a Thin Alumina Nanowire Framework

**DOI:** 10.3390/nano13243169

**Published:** 2023-12-18

**Authors:** Myoung-Kyu Oh, Hyeonju Kim, Prince Gupta, Kyoungsik Kim

**Affiliations:** 1 Advanced Photonics Research Institute (APRI), Gwangju Institute of Science and Technology (GIST), Gwangju 61005, Republic of Korea; cpfl92811@gmail.com; 2 Carnot de Bourgogne Interdisciplinary Laboratory (Laboratoire Interdisciplinaire Carnot de Bourgogne), CNRS UMR 6303, University of Burgundy Franche-Comté (Université de Bourgogne Franche-Comté), 21000 Dijon, France; pri1989.iitr@gmail.com; 3 School of Mechanical Engineering, Yonsei University, Seoul 03722, Republic of Korea

**Keywords:** SERS substrate, aggregated Ag nanowires, dispersed Ag nanoparticles, anodic aluminum oxide

## Abstract

To develop highly sensitive surface-enhanced Raman spectroscopy (SERS) films, various types of aggregated Ag nanowire (NW) and nanoparticle (NP) complex structures were fabricated using anodic aluminum oxide (AAO) templates and thermal evaporation. Aggregated AgNW structures with numerous tapered nanogaps were fabricated via Ag deposition on aggregated thin alumina nanowires of different lengths. AgNP complex structures were obtained by collapsing vertically aligned thin alumina nanowires 1 μm in length and depositing AgNPs on their tops and sides using surface tension during ethanol drying after functionalization. The Raman signal enhancement factors (EFs) of the samples were evaluated by comparing the SERS signal of the thiophenol (TP) self-assembled monolayer (SAM) on the nanostructures with the Raman signal of neat TP. EFs as high as ~2.3 × 10^7^ were obtained for the optimized aggregated AgNW structure (NW length of 1 μm) and ~3.5 × 10^7^ for the optimized AgNP complex structure. The large EF of the AgNP complex film is attributed mainly to the AgNPs dispersed in three dimensions on the sides of the thin alumina nanowires, strongly implying some important, relevant physics yet to be discovered and also a very promising nanostructure scheme for developing ultrahighly sensitive SERS films with EF > 10^8^.

## 1. Introduction

Raman spectroscopy has significant potential for the simultaneous detection of multiple constituents using a simple spectrometer. With the development of novel metal nanostructures, Raman signals can be sufficiently enhanced to enable single-molecule detection and real-time trace-level constituent analysis [1,2]. Surface-enhanced Raman spectroscopy (SERS) is a powerful analytical technique capable of highly sensitive and selective molecular detection [1]. The detection capability of SERS is determined by the Raman signal-enhancing power of metal nanostructures.

A review of recent advances in highly sensitive SERS films reveals that several studies have been conducted on developing SERS films with enhancement factors (EF) > 10^8^ [3,4,5,6,7,8,9,10,11,12,13]. The most promising nanostructure schemes reported thus far have been based on the use of Ag nanorod (NR) array and Ag nanowire (NW) bundle substrates, which can yield EFs > 10^8^. In 2005, Zhao et al. grew AgNR arrays using oblique-angle deposition via physical evaporation [4,5,6,7,14]. When the length, diameter, and density of the AgNRs were ~1 μm, ~100 nm, and 10 μm^−2^, respectively, the substrates exhibited huge EFs higher than 10^8^. In 2016, Zhu et al. fabricated an AgNW bundle array using anodic aluminum oxide (AAO) templates and surface tension between the nanowires during water drying [8]. The array of AgNW bundles yielded an EF of ~10^8^ when the diameter and length of the AgNWs were ~70 and 800 nm, respectively. Research studies on nanostructure schemes supporting EF > 10^7^ have been somewhat scarce until now. In 2012, Huang et al. fabricated an aligned AgNR array using an AAO template and electrodeposition [15,16]. When the space between the AgNRs was ~10 nm, EFs as high as 3 × 10^7^ were observed. In 2022, Sun et al. fabricated a hybrid nanostructure of an AgNP-coated Au nanofunnel array using AAO templates and observed an EF of 2 × 10^7^ [17]. In 2012, Zhai et al. deposited multiple layers of AgNPs on chemically modified agarose films and observed an EF of 1.5 × 10^7^ [18]. From this brief survey, it becomes clear that the use of AgNRs or NWs is a promising scheme for achieving very high EFs and that the use of AAO templates is powerful for developing highly sensitive SERS films. By comparison, 3D hybrid nano–nano or micro–nanostructures have been the main focus of current research for realizing the highest EFs [9,12,17,19,20]. This trend is expected to continue in the near future.

In the authors’ previous study, an Ag-coated aggregated nanowire structure based on an AAO template was a promising SERS medium, exhibiting an EF of ~2 × 10^7^ over a large area and excellent reproducibility [21,22]. From this observation, it is expected that the surface plasmonic effect can be further promoted if the morphological properties are further optimized, such as by increasing the density of the tapered nanogaps.

In this study, aggregated nanowires were exploited to promote SERS activity further. First, various alumina nanowire lengths from 0.6 to 10 μm were tested to determine which would yield the optimized aggregated nanowire structure with the highest EF; Al_2_O_3_ layers of various thicknesses were employed on AAO templates. Second, Ag was deposited on vertically aligned alumina nanowires 1 μm in length, after which nanowire aggregation was performed using surface tension from the drying of ethanol after a functionalization process, which resulted in an AgNP complex structure rather than an aggregated AgNW structure. Third, the thickness of the Ag coating applied via thermal evaporation was optimized for efficient nanogaps or AgNP structures. The morphological properties of the samples were investigated via scanning electron microscopy (SEM) and energy-dispersive X-ray (EDX) spectrometry. The EFs of the nanostructured films were measured by comparing the Raman spectra of neat thiophenol (TP) with the SERS spectra of the TP self-assembled monolayers (SAMs) on the nanostructured surfaces. The highest EF obtained for the aggregated AgNW structures was ~2.3 × 10^7^, which was achieved using Ag-coated aggregated nanowires 1 μm in length, whereas that for the AgNP complex structure was ~3.5 × 10^7^. Micro-Raman measurements of the AgNP complex structure showed that the large EF could be attributed mainly to the AgNPs distributed on the sides of the thin alumina nanowires.

## 2. Materials and Methods

### 2.1. Materials

**Figure 1:**
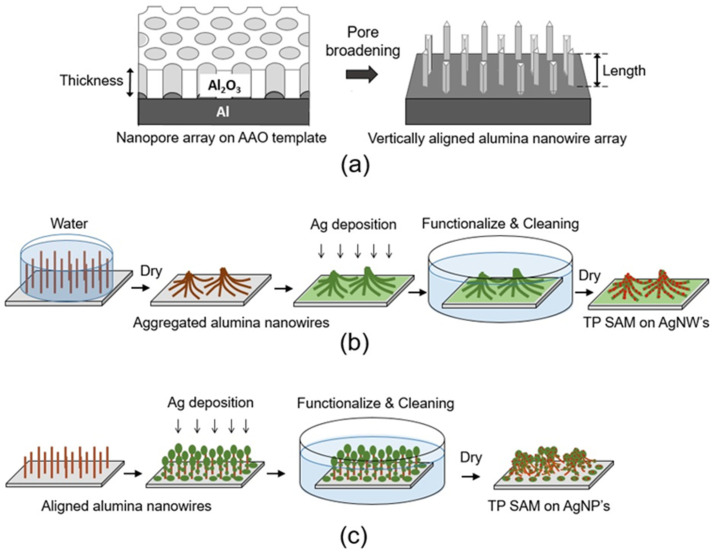
Schematics of the whole fabrication process. (**a**) Vertically aligned alumina nanowire array fabrication from nanopore array on anodic aluminum oxide (AAO) template. (**b**) Aggregated Ag nanowire (AgNW) fabrication followed by functionalization by thiophenol (TP). (**c**) Ag nanoparticle (AgNP) complex structure fabrication utilizing surface tension during ethanol drying after functionalization by TP.

For the aggregated nanowire structures, vertically aligned alumina nanowires were fabricated via the pore broadening of AAO templates [21,22], as schematically explained in Figure 1a. To control the lengths of the nanowires, AAO templates of different thicknesses were employed. Nanopores 30 nm in diameter, forming a precise array of equilateral triangle unit cells, the side length of which was 100 nm, were initially prepared in the AAO templates as follows: First, anodization was performed for 15 h in 0.3 M oxalic acid at a constant voltage of 40 V, at 10 °C, following an etching of the alumina layer in 6 wt% chromic acid for 5 h at 80 °C. Subsequently, a second anodization step was performed for 1 h to obtain AAO layers containing well-aligned nanopores. The nanopores in the AAO layers obtained in this way were enlarged to merge and result in vertically aligned nanowire arrays with a triangular cross section after the pore broadening process via chemical wet etching with 5 wt% phosphoric acid at 40 °C for ~16 to 18 min. If washed with water, the nanowires were aggregated by the water–air surface tension between them during the drying process. Because the surface tension was in random directions, the self-aggregated nanowire bundles displayed irregular three-dimensional (3D) patterns similar to mountain ridges and valleys (Figure 1b). However, the self-aggregated nanowire bundles exhibited different patterns depending on the nanowire length. For example, 5 μm nanowires typically result in microfunnel arrays with mountain ridges and valleys. Each microfunnel structure comprises hundreds of nanowires. Furthermore, 1 μm nanowire bundles lead to sub-micron-scale isolated nanowire bundle structures composed of tens of nanowires. The vertically aligned nanowire array structure obtained after pore broadening can maintain its morphology without collapsing when the fabricated nanowire array sample is treated differently. To prevent the nanowire from collapsing from surface tension, the nanowire template was dipped in an isopropyl alcohol solution and dried in a chamber with supercritical conditions using carbon dioxide at 80 °C under 13.5 MPa pressure (Figure 1c).

Ag was deposited onto the nanostructured films via thermal evaporation to form metal nanowire structures. Ag wires of >99.99% purity were employed as deposition material, and the base pressure of the thermal evaporator was maintained below 5 × 10^−6^ Torr during the deposition processes. Various deposition amounts were tested to determine the optimal Ag coating thickness for achieving the highest SERS activity.

### 2.2. Characterization Methods

The morphological properties of the fabricated nanostructures were analyzed via field-emission SEM (Hitachi, Tokyo, Japan, S-4700) and EDX (Hitachi, Tokyo, Japan, S-4800; Horiba, EMAX).

To assess the EFs of the fabricated SERS films, TP SAMs were prepared on the surfaces of the films. For functionalization by TP, the samples were dipped in a 15 mM ethanolic solution of TP for 24 h at room temperature. The substrates were carefully rinsed with copious amounts of ethanol to remove any physically adsorbed thiol molecules. Finally, the samples were dried using a stream of N_2_ gas.

A micro-Raman spectrometer employing a specialized spectroscopy fiber bundle, a 785 nm pump laser, and a spectrometer (SOL Instruments, Minsk, Belarus, MS3504i) was used for optical measurements. The pump laser power on the sample during the measurements was 10 mW. An aspheric lens with a numerical aperture > 0.5 and a working distance of 8 mm was placed in front of the SERS film to focus the pump laser on the SERS film and collimate the reflected Raman signal simultaneously. The spot size of the pump laser on the SERS film was made as large as 30 μm in diameter to protect the SERS film.

Another micro-Raman spectrometer was used to separate the contributions to the observed SERS signal intensities spatially. A 543 nm pump laser (CNI laser, Changchun, China, MLL-FN-543 nm-20mW) and an objective lens with a numerical aperture of 0.4 were employed. For the acquisition of Raman spectra, an imaging spectrometer 50 cm in focal length (TPI, Pennsauken, NJ, USA, SP2500i) and a cooled Si-CCD array detector (Andor, Belfast, Northern Ireland, UK, DU401A-BV) were used. The SERS peak heights at 1004 cm^−1^ were recorded on different focal positions of the nanostructured film functionalized by TP. The input power of the pump laser was set to 10 μW, and the acquisition time for each measurement was 30 s. From experimental parameters, the actual spot diameter of the pump laser on the nanostructured film was estimated to be 1–3 μm.

## 3. Results and Discussion

### 3.1. Ag Nanowire Structures

**Figure 2:**
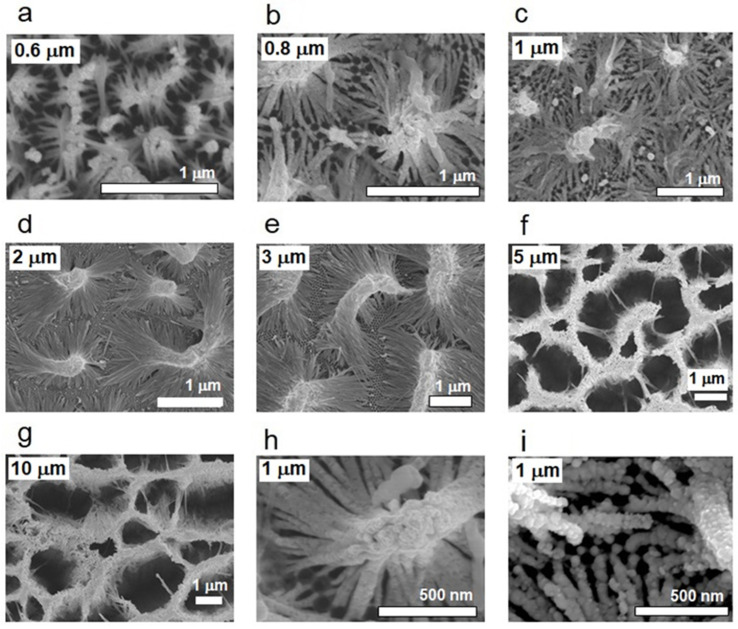
Scanning electron microscopy (SEM) images of aggregated nanowire structures of various nanowire lengths: (**a**) 600 nm, (**b**) 800 nm, (**c**) 1 μm, (**d**) 2 μm, (**e**) 3 μm, (**f**) 5 μm, and (**g**) 10 μm. Precise images of aggregated nanowires 1 μm in length with Ag coating thickness of (**h**) 60 and (**i**) 100 nm. Scale bar in each image corresponds to (**a**–**g**) 1 μm and (**h**,**i**) 500 nm.

Figure 2 shows SEM images of various aggregated nanowire structures fabricated via nanopore broadening and nanowire aggregation processes from AAO templates. Figure 2a–g depict nanowire bundles of different nanowire lengths coated with an Ag coating 60 nm in thickness. Nanowires of various lengths, namely, 600 nm, 800 nm, 1 μm, 2 μm, 3 μm, 5 μm, and 10 μm, were fabricated and employed to develop an optimized nanowire bundle structure for SERS. From the SEM images, the morphologies of the nanowire bundle structures were found to be very specific and exhibit a trend with respect to the nanowire length. When the nanowire length is shorter than 2 μm, isolated nanowire bundling with one top in each structure can be observed. Because the center-to-center distance between neighboring nanowires is fixed at ~50 nm, numerous tapered nanogaps appear near the bottom of each bundle structure. The morphological properties of the tapered nanogaps seem to depend on the length of the nanowires over the entire scale. The number and inclination angles of the nanowires in each bundle structure were found to increase as the nanowire length increases. When the nanowire length was longer than 3 μm, a mountain-ridge-like structure started to appear from the extension of the bundle top composed of numerous nanowires. With longer nanowires, the inclination angle of each nanowire and the coverage of the ridge structure composed of conglomerated nanowires are relatively large, which is not recommended for high EF. Nonetheless, the most important factor determining the EF should be the behavior of tapered nanogaps around the bottom, which induces a hot spot effect. The most recommended morphological property for high EF should guarantee more hot spot sites and a greater chance for the incident light to reach the hot spot area, where the nanowire length can be a crucial factor. Nanowires shorter than 600 nm were excluded from this experiment because the expected surface plasmon effect of the nanowire bundles of these short nanowires was limited, and the fabrication of nanowire bundle structures using such short nanowires did not proceed well.

**Figure 3:**
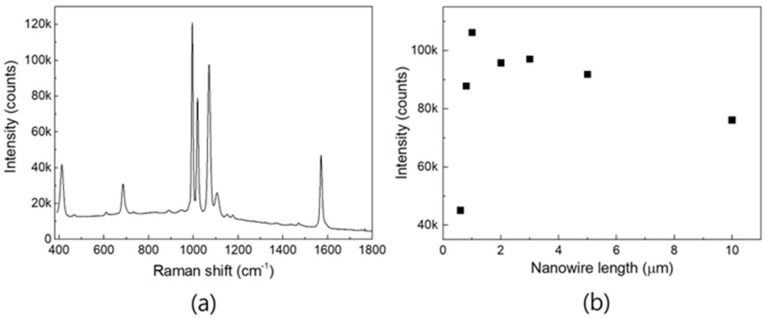
(**a**) Surface-enhanced Raman spectroscopy (SERS) spectrum of TP self-assembled monolayer (SAM) on structure composed of Ag-coated aggregated nanowires 1 μm in length with a coating thickness of 100 nm. Acquisition time was 1 s. (**b**) SERS signal intensities of peaks at 1004 cm^−1^ with respect to nanowire length for Ag coating thickness of 100 nm.

Figure 3a shows the SERS spectrum of the TP SAM on an Ag-coated nanowire 1 μm in length when the Ag coating thickness is 100 nm. Hereafter, all given spectra are not intensity-corrected. The acquisition time for the spectra was 1 s. The graph in Figure 3b shows the relative signal intensities of the SERS peaks at 1004 cm^−1^ with respect to the nanowire length. Even though the observed morphologies of the nanowire structures were very specific and dependent on nanowire length, as in Figure 2, the SERS activities of the aggregated nanowires 0.8 to 5 μm in length had no special difference. From this observation, the optimized nanowire length for the aggregated nanowire structure was determined to be between 0.8 and 5 μm, of which 1 μm seems to be preferable. This trend is inferred to have occurred because the density, scale, and coverage of the nanogaps were nearly the same as those of the aggregated nanowire structures shown in Figure 2b–f.

**Figure 4:**
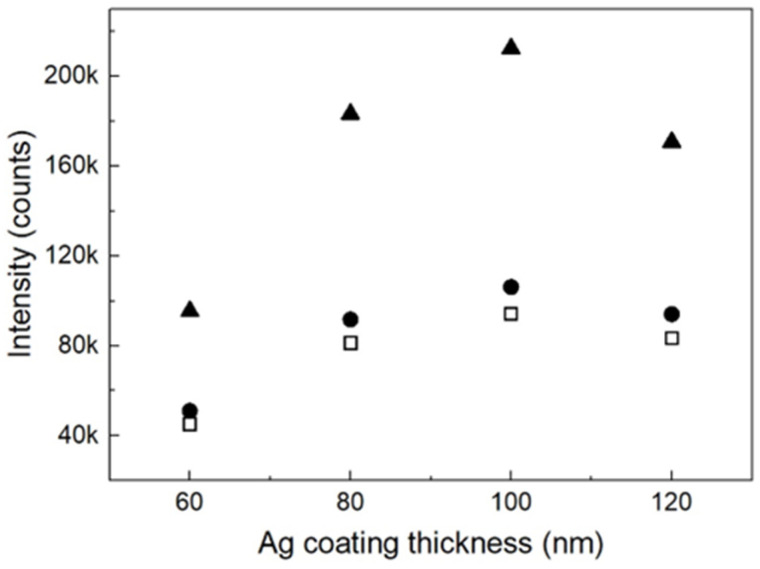
Signal intensities of TP SERS peak at 1004 cm^−1^ for various Ag coating thicknesses. Filled circles and open squares represent data acquired for structures of aggregated nanowires 1 and 5 μm in length, respectively. Filled triangles represent data for nanowires 1 μm in length with Ag deposition on vertically aligned nanowires and subsequent aggregation of the nanowires by functionalization.

The experimental results visualized in Figure 4 show that the optimum Ag coating thickness is ~100 nm, irrespective of the nanowire length. Here, the signal intensity was also measured based on the TP SERS peak at 1004 cm^−1^. This observation can be understood as an indication that the scales of the nanogaps around the bottom were optimized, and that the metal coating on the nanowire surfaces around the nanogaps was uniform with a deposition amount of 100 nm because the very complicated morphology of the bare alumina nanowire structures requires a sufficient amount of deposition for a uniform Ag coating. This assumption was proven by EDX analyses on the samples. In conjunction with the elemental analysis data (Table S1), the elemental mapping data in Figures S1 and S2 show that there were many sites where the amounts of Ag deposition were much lower than the average. Figure 2h,i provide precise morphologies of nanowires 1 μm in length around the bottom as a representative example, shown in SEM images of the aggregated nanowires with coating thicknesses of 60 nm and 100 nm, respectively. Based on these images, the average diameters of the Ag nanowires in the Ag-coated samples with coating thicknesses of 60 and 100 nm were ~30 and 65 nm, respectively. Notably, the optimized nanoparticle size and nanowire thickness were 50–100 nm. The increased surface roughness of the nanowires in the 100 nm coated sample should have produced another positive effect on the SERS activity of the nanostructure. Importantly, the nanogap scales should be optimized by the increase in nanowire thickness and surface roughness of the Ag-coated sample with a coating thickness of 100 nm.

### 3.2. Ag Nanoparticle Structures

**Figure 5:**
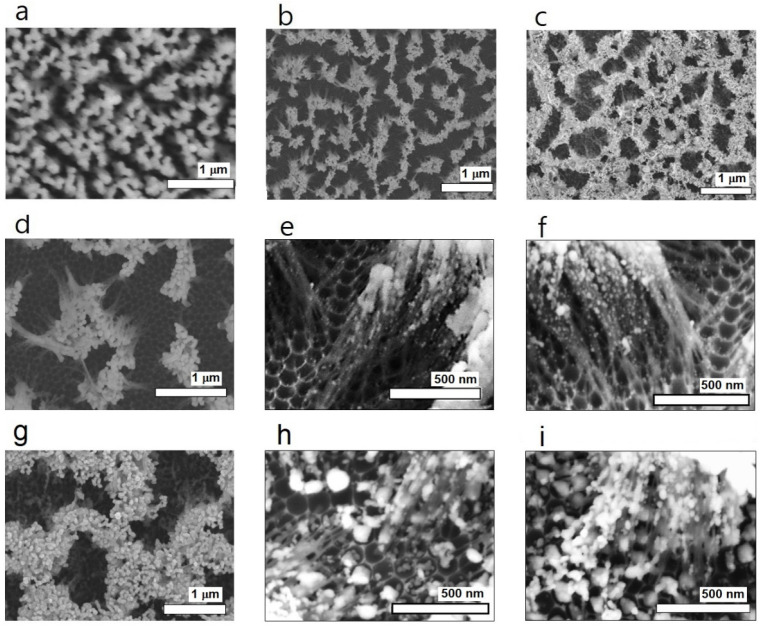
SEM images of nanowires 1 μm in length (**a**) after Ag deposition on vertically aligned nanowires to a thickness of 100 nm and (**b**–**i**) subsequent aggregation of the nanowires by functionalization. (**b**,**d**,**e**,**f**) Aggregated nanowire structures with Ag deposition thicknesses of 60 nm and (**c**,**g**,**h**,**i**) 100 nm. Scale bars correspond to (**a**–**d**,**g**) 1 μm and (**e**,**f**,**h**,**i**) 500 nm.

Vertically aligned alumina nanowire arrays, which are obtainable via the supercritical drying of isopropyl alcohol, can also aggregate during the drying of ethanol after functionalization (Figure 1c). In this study, the vertically aligned alumina nanowires were coated with Ag before the functionalization process. Figure 5a shows an SEM image of the vertically aligned nanowires after Ag deposition to a thickness of 100 nm. Ag nanoparticles were formed on top of the nanowires depending on the amount of Ag deposition. When the Ag deposition thickness was 60 nm, Ag ellipsoid structures with short- and long-axis cross-sectional diameters of ~70 and ~100 nm, respectively, were observed. An Ag tail with an extension of ~100 nm was also formed under the ellipsoids. Consequently, the AgNPs on top of the nanowires were in the shape of matched heads. As the Ag deposition proceeded, AgNPs also grew on the sides of the vertically aligned nanowires and on the bottom of the AAO substrate. When the thickness of the deposited Ag was 100 nm, the diameters of the Ag ellipsoids along the short and long axes were 70–80 and 150 nm, respectively. For comparison, when the thickness of the Ag deposition was 60 nm, the AgNPs deposited on the sides of the alumina nanowires were located mostly in the upper half of the nanowires (Figure 5e,f). Moreover, the sizes of the AgNPs ranged from several nanometers to 30 nm. By contrast, when the thickness of the Ag deposition was increased to 100 nm, the AgNPs were distributed on the entire nanowire and on the bottom of the template. Furthermore, the diameters of the Ag nanoparticles on the alumina nanowires mainly ranged from 30 to 100 nm. At the bottom, AgNPs ~90 nm in diameter were grown in nearly all nanopores of the AAO template. After functionalization, the nanowires collapsed to form aggregated nanowire structures, which depended on the amount of Ag deposited. Figure 5b,d–f show aggregated nanowire structures after Ag deposition to a thickness of 60 nm. The bright regions in the images depict the aggregated AgNPs on top of the alumina nanowires. Owing to the surface tension during the drying process, the AgNPs were strongly packed within the alumina nanowire framework. Furthermore, Figure 5c,g–i show SEM images of AgNPs on aggregated alumina nanowires after Ag deposition to a thickness of 100 nm. According to Figure 5b,c, the average coverages of the bright region or AgNPs on top of the alumina nanowires were ~30% and ~50%, respectively, which are attributed mainly to the sizes of these AgNPs. The deposition of such AgNPs on nanowires should be employed in developing highly sensitive SERS substrates [18]. However, the EF value for this type of nanostructured scheme can hardly be found in the literature. The most notable findings from this experiment are that thin and long (1 μm) nanowires should be employed and that the medium and bottom regions of the nanostructure are fully exposed owing to the extreme collapse of the alumina nanowires due to strong surface tensions between neighboring nanowires. The EDX data for these AgNP complexes support the aforementioned analyses of the morphological properties using the SEM images (Table S2, Figures S3 and S4).

The dependence of SERS signal intensities on the amount of Ag deposition is shown in Figure 4. The filled triangles represent data for AgNPs on aggregated alumina nanowires. Compared with the structures composed of Ag-coated aggregated nanowires 1 μm in length (Figure 2c,i), this AgNP nanostructure had a ~1.5 times higher EF value, exhibiting the highest signal intensity among the aggregated nanowire samples given in Figure 2. Further, the AgNP nanostructure with an Ag deposition thickness of 100 nm exhibited an EF value that was approximately 2.5 times higher than that of the AgNPs with an Ag deposition thickness of 60 nm. With regard to the morphological properties related to this observation, the AgNPs in three different regions, namely, the plane roof, slope, and plane bottom of the microscale mountain ridge and valley structures of the aggregated alumina nanowires need to be considered separately. First, with regard to the plane roof, nanostructures of closely packed ellipsoid AgNPs formed therein are owing to the strong surface tension of the tightly aggregated alumina nanowires. This type of nanostructure is characterized by numerous nanogaps and, therefore, can support an EF higher than 10^7^. Notably, for the samples with Ag deposition thicknesses of 60 and 100 nm, the thicknesses of the AgNPs were ~70 and 70–80 nm, respectively. These are the optimum particle sizes for Raman signal enhancement. Second, with regard to the slope, the AgNPs therein were distributed with a high density on the thin alumina nanowire framework. In view of the hot spot effect, this AgNP nanostructure is not expected to exhibit a higher enhancement than that of the AgNPs on the plane roof because nanogaps narrower than several nanometers are sparse in this structure. Third, with regard to the bottom, no special AgNPs were observed therein for the samples with an Ag deposition thickness of 60 nm, whereas for the samples with an Ag deposition thickness of 100 nm, AgNPs ~90 nm in diameter had grown in nearly every nanopore of the AAO template. Nonetheless, the contribution from this bottom nanostructure should be negligible because its enhancement factor should be lower than that of the roof region by more than one order of magnitude.

The main characteristic of AgNP nanostructures in the slope region is that AgNPs of various sizes are dispersed in three dimensions (3D) on the aggregated alumina nanowires. For the samples with an Ag deposition thickness of 60 nm, the diameters of the AgNPs ranged from several nm to 30 nm, and the 3D dispersion region existed mainly in the upper half of the aggregated alumina nanowires. The coverage of this slope region exposed to the incident laser was ~10%. For comparison, the coverage of the bottom region was ~60%. Furthermore, for the samples with an Ag deposition thickness of 100 nm, the sizes of the AgNPs ranged mainly from 30 to 100 nm, and the AgNP distribution region was from the bottom to the place immediately under the AgNPs at the top. Moreover, the coverage of the slope region exposed to the incident laser was ~40%; for comparison, the coverage of the bottom region was ~10%. Given the hot spot effect, these AgNP bulk nanostructures consisting of numerous AgNPs of different sizes dispersed in 3D on the thin alumina nanowire framework are not expected to exhibit higher Raman signal enhancements than those of the closely packed AgNPs on the roof region. However, despite these findings, it was decided that any conclusions on the enhancement factor for this type of dispersed AgNPs in 3D should be delayed until some important evidence is obtained because this is probably the first time that such AgNP nanostructure schemes have been experienced. This implies it cannot yet be concluded if the contribution of the roof region to the observed Raman signal enhancement is dominant or comparable to that of the AgNPs in the slope region.

### 3.3. Raman Signal Enhancement Factor

**Figure 6:**
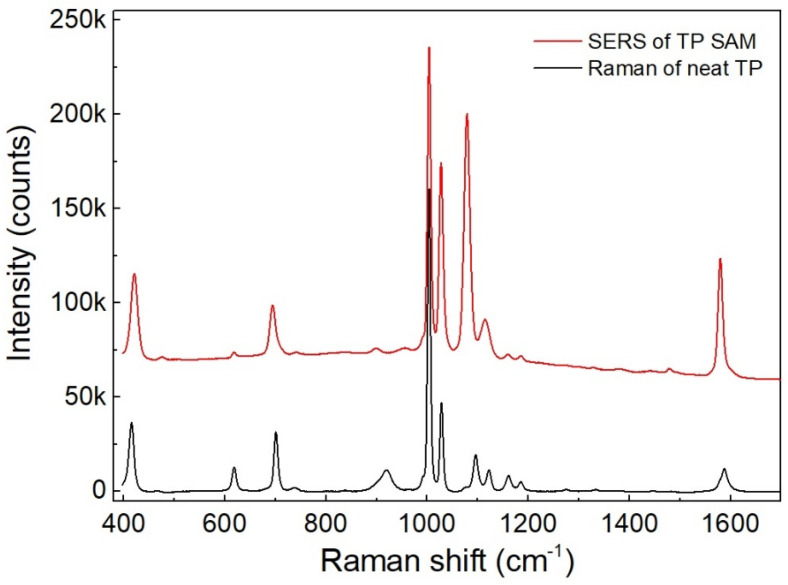
SERS spectrum of TP SAM on AgNPs on aggregated nanowire structures (Figure 5c,g–i) with an acquisition time of 1 s (red). Reference Raman spectrum of neat TP with a thickness of 1 mm and acquisition time of 30 s. Raman spectrum was multiplied by 2.3 to match the heights of the Raman and SERS peaks at 1004 cm^−1^ (black). SERS spectrum is vertically shifted for convenient comparison.

Figure 6 shows the SERS spectrum of the TP SAM on the AgNPs on the aggregated nanowires of 1 μm length given in Figure 5c,g–i, in comparison with the reference Raman spectrum of neat TP (99%). The acquisition time of the SERS spectrum was 1 s, and that of the Raman spectrum was 30 s. To match the heights of the Raman and SERS peaks at 1004 cm^−1^, the Raman spectrum was multiplied by 2.3. In this way, the SERS and Raman spectra were normalized with respect to the peak at 1004 cm^−1^.

The EF of a SERS substrate is determined by the relation

(1) $$EF=\frac{I_{SERS}/I_{Raman}}{N_{SAM}/N_{bulk}}\;,$$

where *I*_SERS_ and *I_Raman_* represent the intensities of the SERS and Raman signals, respectively, whereas *N*_SAM_ and *N_bulk_* are the numbers of probe molecules in the SAM and bulk states contributing to the signals, respectively.

The numerator of Equation (1) can be calculated by comparing the signal intensities of the SERS and Raman spectra shown in Figure 6. At first, the line shape of the SERS spectrum of the TP SAM seems similar to that of the Raman spectrum. However, a more detailed examination would reveal that the SERS peaks at 1026, 1078, and 1575 cm^−1^ are higher than their corresponding Raman peaks at 1027, 1095, and 1583 cm^−1^ by 2.1, 7.7, and 5.3 times, respectively. Moreover, notable frequency shifts, such as −1, −15, and −8 cm^−1^ from the Raman to SERS peaks, were found in these three SERS and Raman peak pairs. Frequency shifts were also observed for the other SERS peaks. The observed frequency shifts of 6 cm^−1^ at 420 cm^−1^, −5 cm^−1^ at 695 cm^−1^, and −8 cm^−1^ at 1111 cm^−1^ correspond to the relative signal enhancement factors of 1.66, 1.1, and 1.9, respectively. Even though a proportional relationship was not found, the frequency shift and relative signal enhancement seemed to be closely related. Therefore, the frequency shift can be a basic indicator of the relative Raman signal enhancement. The aforementioned six peaks are among the seven prominent peaks in the SERS spectrum. Therefore, the line-shape change in the SERS spectrum from that of the Raman spectrum can be described by the relative signal enhancements and relevant frequency shifts in these six prominent SERS peaks. Furthermore, the SERS peak at 1004 cm^−1^, the most prominent one in both the SERS and Raman spectra, notably does not exhibit any frequency shift and linewidth broadening. This is the reason for normalizing the SERS and Raman spectra with respect to this peak.

These observations can be explained by the chemical enhancement (CE) accompanying the large electromagnetic enhancement (EE) [23,24,25]. Therefore, the Raman signal enhancement at each peak was derived from the relationship EF = EE × CE. According to previous studies [24], there are two types of CEs: dynamic and static. Dynamic CE, which is generally known as CE, arises from an increase in population difference between the two ground states of a Raman transition, to which charge transfer from a metal surface to adsorbed molecules induced by an incident laser field is attributed. When this occurs, the spectrum line shapes can change dramatically. By contrast, the static CE is due to charge transfer from a metal surface to adsorbed molecules by chemical bonding between S and metal atoms, irrespective of an incident laser. Compared with dynamic CE, static CE is localized around the S atom, and the degree of enhancement is limited. Nonetheless, both CEs accompany vibration mode frequency shifts because any kind of charge supply changes the coupling constant between atoms.

Given these CE mechanisms, the aforementioned observations can be explained in a basic manner, and the SERS peak at 1004 cm^−1^ is reasonably believed to be free of CE or experience a CE close to 1 [24]. Therefore, the SERS peak at 1004 cm^−1^ can be the only one reliable reference for EF evaluation out of the seven prominent peaks in the SERS and in the Raman spectra. Consequently, the value of *I*_SERS_/*I_Raman_* for evaluating EF is 70 using the SERS and Raman peaks at 1004 cm^−1^.

Further observations on the SERS spectrum shown in Figure 6 reveal linewidth broadenings by a factor of 1.4 to 1.8 for the prominent SERS peaks at 420, 695, 1078, and 1111 cm^−1^ and relative enhancements of 0.16, 0.27, and 0.7 for the peaks at 618, 1160, and 1185 cm^−1^, respectively. Compared with these, the Raman peak at 917 cm^−1^, which does not have its corresponding SERS peak, is associated with the C–S–H bend [26]. These linewidth broadenings and relative signal decreases are not trivial and should be explained, where other mechanisms, such as the dependence of vibration modes on the orientation of adsorbed molecules on a metal surface rather than the CE, can play important roles [27]. These mechanisms can be investigated in future studies. Polarization effects and lineshape effects of localized surface plasmon resonance should also be considered for appropriate spectral analyses. However, in this experiment, these effects were estimated to be negligible in the TP monolayer on the Ag nanostructure systems and thus should be accounted for in later research. Additionally, there remains a probability of having to adjust further the EF obtained from exclusively using the SERS and Raman peaks at 1004 cm^−1^ because the possible dominance of parallel or perpendicular polarization on every surface in a nanostructure can produce some average effect on the Raman signal enhancement with aligned molecules, although if it existed, the correction factor is expected to be less than two. Nonetheless, this should also be considered in future studies.

For *N*_SAM_, the focal spot diameter of the pump laser, which is ~30 μm, and the known surface number density of TP SAM on a flat Au surface, which is 4.7 nm^−2^, can be employed [28]. Considering that the actual surface area of the SERS film may be several times broader than that of a flat surface based on the morphologies shown in Figure 5, the actual surface number density of the TP SAM on the nanostructure can also be reasonably assumed to be lower than that on a flat surface by several times. Therefore, the use of a surface number density on a flat surface does not make any meaningful difference. A detailed discussion of this is provided in Section 3.4. Consequently, the number of TP molecules contributing to the SERS signal was derived to be ~4 × 10^9^. Furthermore, for *N_bulk_*, the focal spot diameter of the pump laser, which is ~30 μm, and the axial length of the TP solution covered by the Raman spectrometer, which is ~420 μm out of the 1 mm quartz spectroscopy cell, as confirmed by comparing with the signal intensity from a 17 μm thick TP solution, can be used. Using the volume number density of the neat TP, 5.9 × 10^9^ μm^−3^ from a molecular weight of 110 g mole^−1^ and density of 1.07 g cm^−3^, *N_bulk_* was calculated to be ~2 × 10^15^. Therefore, *N*_SAM_/*N_bulk_* is ~2 × 10^−6^. Consequently, an EF value of ~3.5 × 10^7^ was obtained from Equation (1). This EF is ~1.5 times higher than 2.3 × 10^7^, which is the EF of the optimized aggregated AgNW structure employing alumina nanowires 1 μm in length (Figure 2c,i) and calculated using the data in Figure 3. Compared to representative nanostructure schemes with EFs > 10^7^, the nanostructure schemes proposed in this study seem highly competitive (Table 1).

Based on the SERS signal intensities measured using the peak height at 1004 cm^−1^ on various positions within the SERS films, the relative standard deviation (RSD) of the Ag aggregated nanowire structure in Figure 2c,i is <5%, whereas that of the AgNP complex in Figure 5c,g–i is <3%, implying that the SERS films have excellent reproducibilities. The stabilities of the fabricated nanostructures as SERS media were also investigated using the SERS signal intensities of the samples, which were continuously illuminated by a 785 nm laser with a power of 10 mW and diameter of 30 μm. For the aggregated AgNW samples, no SERS signal changes were observed. By contrast, for the AgNP complex samples, the SERS signal decreased by ~20% after 10 min of illumination. The confirmed stability of the aggregated AgNW samples is attributed to the sound aggregation of alumina nanowires due to strong surface tension and the Ag film covering them. By contrast, the AgNP complex samples were obtained via the aggregation of alumina nanowires with the deposition of AgNPs on their sides and tops. No additional mechanical support schemes exist for this type of nanostructure. This intrinsic mechanical vulnerability led to a long-term SERS signal decrease. Nonetheless, even with this degree of vulnerability, the AgNP complex substrates exhibited great SERS signal stability within minutes, and there seem to be various methods for circumventing this weakness in their long-term stability.

### 3.4. On the Surface-Enhancement Factor (EF) Assessment Schemes

Based on previous studies, two types of schemes have been employed in EF assessment: one uses a monolayer on nanostructures (monolayer scheme), and the other uses a given number of adsorbed probe molecules on the nanostructures (drop-and-dry scheme). The advantage of a monolayer scheme is that robust and reliable assessment can be performed with it, whereas the advantage of the drop-and-dry scheme is that the number of probe molecules can be determined, allowing for prompt and convenient assessments. Of course, the two schemes can harmonize well under well-defined conditions. However, there are some problems regarding the appropriateness of these assessment schemes, e.g., with regard to whether it is appropriate to use the surface number density of a monolayer on a flat surface and with regard to whether the EF should be assessed based on the number of probe molecules on a nanostructure.

On these problems, the authors concluded that the monolayer scheme was appropriate and that the usage of the surface number density of a probe molecule monolayer on a flat surface was also appropriate. At first glance, the drop-and-dry scheme seemed appropriate according to Equation (1). However, Equation (1) is not for the average EF but, rather, for a single EF of an infinitesimal area. On every nanostructured surface, it is highly unlikely for the incident light intensities to be equal at all points. Therefore, Equation (1) should be replaced with an integral formula, as explained by Le Ru et al. [29]. To demonstrate, when a nanostructured surface is tilted by *θ*, the illuminated area becomes *A*/cos *θ*, and the incident light intensity becomes *I*_0_·cos *θ*, where *A* and *I*_0_ are the cross-sectional area and mean light intensity of the incident light, respectively. The Raman signal is independent of the tilt angle, *θ*, only when a constant surface number density of probe molecules is assumed. Based on common sense, this picture is evidently appropriate because the surface properties of the nanostructured surface represented by EF do not change. The constant surface number density of the probe molecules can properly reflect the surface properties, such as the Raman signal enhancement factor. Additionally, obtaining the probe molecule number from the cross section of the incident laser beam and multiplying it by the average surface number density on a flat surface is appropriate from both practical and theoretical points of view. Every nanostructure has an intrinsically curved surface. Usually, the actual surface area of a nanostructure is 3–6 times larger than that of a flat surface for a given cross section of the incident laser beam. The variation in the actual surface area with respect to the nanostructure schemes was much lower than expected. If there is no reflection on the nanostructured surface, the enhanced Raman signal on any nanostructured surface is the same as that on a flat surface. However, a certain type of gain based on curved surfaces is usually obtained because some degree of reflection exists on nanostructured surfaces. Therefore, the number of probe molecules from the cross-sectional area of the incident laser beam multiplied by the surface number density of the monolayer is conceptually appropriate. This scheme simplifies the EF assessment process, irrespective of the specific nanostructure.

Furthermore, in the context of the drop-and-dry scheme, an important problem can be raised on the spatial uniformity of probe molecules adsorbed on nanostructures. However, this problem can be confirmed only through practical experience. When a 1 μL drop of probe molecule solution is dispersed on a 1 cm^2^ area, the actual surface number density of the probe molecules is ~0.03 monolayer for a concentration of 10^−^^4^ M, reflecting the actual surface area of a nanostructure. Then, 10^−^^4^–10^−^^5^ M is an appropriate concentration for EF evaluation. Meanwhile, it is also noticeable that surface-enhanced Raman signal intensities consistently tend to be nonproportional to the number of probe molecules in drop-and-dry SERS measurements [3,6,9,10,12,13], which clearly contradicts Equation (1). To demonstrate, when the concentration changes from 10^−4^ to 10^−10^ M, the Raman signal intensity decreases by 3–4 orders of magnitudes, which is beneficial in view of trace constituents detection in liquids but indicates that the distribution of probe molecules adsorbed on a nanostructure is not uniform and that the degree of probe molecule concentration in nanogaps will increase as the probe molecule concentration decreases. This phenomenon is attributed to the behavior of probe molecules during the drying process. The bottom and gap areas of nanostructures are promising candidate sites for probe molecule concentration because they are the last places where the solution remains during the drying process.

### 3.5. Roof Versus Slope Contributions

As discussed in Section 3.2, the main contribution to the observed Raman signal enhancement, 3.5 × 10^7^, must be attributed to experimental evidence.

**Figure 7:**
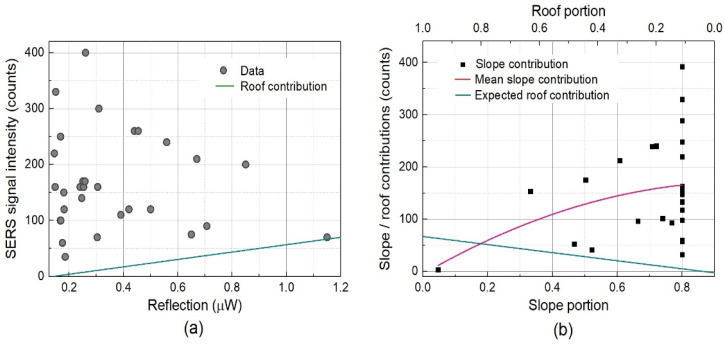
Experimental data and analysis results from micro-Raman spectroscopy on a AgNP complex film with 100 nm Ag deposition. The heights of SERS peaks at 1004 cm^−1^ of TP SAM on the nanostructure were used for SERS signal intensities. (**a**) SERS signal intensity data dependent on the measured pump laser reflection signal output. The green line shows the expected contribution to the SERS signal intensity from the roof. (**b**) Calculated slope contribution to the measured SERS signal intensities with respect to slope portion. The red and green lines show the mean slope and expected roof contributions with respect to their portions.

A micro-Raman spectroscopy was performed to separate the observed Raman signals into two regions, specifically the plane roof and slope regions. The objective of this experiment was to confirm the contribution of the roof region. Based on the SEM images in Figure 5, the reflection in the roof region is much higher than that in the slope and bottom regions, and the optical properties of the roof region, such as reflectance and SERS activity, are reasonably assumed to be homogeneous. Figure 7a shows the measured SERS signal intensities in conjunction with their corresponding surface reflections. It should be emphasized that the observed SERS signal was not proportional to the surface reflection, clearly indicating that the contribution from the slope region was comparable to or higher than that from the roof region. The data point corresponding to 1.15 μW of reflection is a rare datum measured on the entire roof region under ideal focusing conditions. Here, the ideal reflection from the roof area is assumed to be 1.2 μW. Based on the homogeneous properties of the roof region, the SERS signal intensity was reasonably assumed to be proportional to the reflection power, as indicated by the green line in Figure 7a. Figure 7a clearly shows that the contribution from the slope region is much higher than that from the roof region in the low-reflection region of the graph. Based on average coverages for the roof, slope, and bottom regions of ~0.5, ~0.4, and ~0.1, respectively, the horizontal axis of the graph in Figure 7a was changed to reflect portions of the slope and roof regions. In Figure 7b, the red line represents the mean slope contribution to the observed SERS signal intensities, whereas the green line represents the roof contribution dependent on the roof portion. For instance, the average contribution from the slope region is ~2.5 × 10^7,^ and that of the roof is ~10^7^ when the slope and roof portions are 0.4 and 0.5, respectively, which are the real parameters of the SERS film. We also noted that, in the high-coverage region of the slope, very high signal intensities occurred among the various signal intensities with wide variation. This indicates that a much higher EF > 108 can be obtained using a well-built AgNP nanostructure block. 

### 3.6. Proposition of New AgNP Nanostructure Scheme

**Figure 8:**
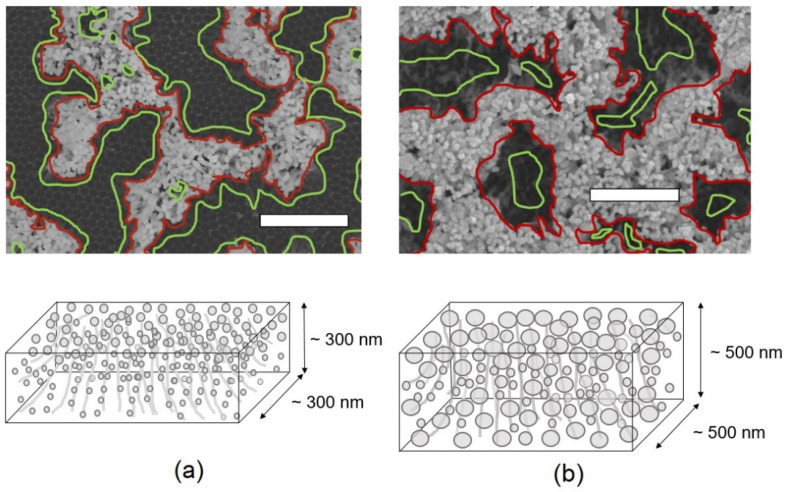
Division of Ag nanoparticle structures of aggregated nanowires 1 μm in length into three parts: roof, slope, and bottom. Regions surrounded by red lines are roof regions, between red and green lines are slope regions, and those surrounded by green lines are the bottom region. Ag deposition thicknesses are (**a**) 60 nm and (**b**) 100 nm. Schematics of Ag nanoparticle volumes in slopes and under roofs are given in lower parts. Grey circles represent AgNPs and grey lines are alumina nanowires. Scale bars (white bars in the figures) correspond to 1 μm.

The observations were summarized as follows: The observed average EF was separated into ~2.5 × 10^7^ from the slope and ~10^7^ from the roof. Under the assumption of full coverage, the EF of the slope was ~6 × 10^7,^ and that of the roof was ~2 × 10^7^. Moreover, the AgNPs on the roofs of the aggregated alumina nanowires with Ag deposition thicknesses of 60 and 100 nm were reasonably assumed to have nearly the same optical properties. For the AgNPs on the aggregated alumina nanowires with an Ag deposition thickness of 60 nm, based on these coverages, the contribution from the roof was ~6 × 10^6,^ and that from the slope was also ~6 × 10^6^ out of the measured EF of ~1.2 × 10^7^. Further, under the assumption of full coverage, the expected EF of the sloped AgNP nanostructure was ~6 × 10^7^, and that of the roof was ~2 × 10^7^. Considering that the AgNPs deposited on the plane surface with the sizes and densities of the AgNPs shown in Figure 5e,f can support limited EF (<10^6^), a completely new picture is needed to explain the observed anomalously high EFs for the AgNPs distributed in 3D. In the lower schematics of Figure 8, the main morphological properties of the AgNPs in the sloped regions are described. Although aggregation of AgNPs was also observed among the AgNPs in the slope region, the degree of aggregation was not comparable to that in the roof region. The sizes of the AgNPs and the dimensions of the bulk were estimated from the roof and slope regions, as shown in Figure 8. With regard to the roof contributions to the observed EF, the highest levels among the EFs reported for the AgNP nanostructures were ~10^7^ and ~6 × 10^6^ for the AgNP structures with Ag deposition thicknesses of 100 and 60 nm, respectively. Clearly, the closely packed AgNPs with sizes of 50–100 nm exhibited the highest EFs among the various AgNP nanostructure schemes.

AgNPs dispersed in 3D on the thin alumina nanowire framework can also be considered. When the size of the AgNPs and separation distances are optimized, EFs of 10^8^–10^9^ are expected for the new AgNP nanostructural scheme.

## 4. Conclusions

In this study, highly sensitive SERS films were fabricated using an advanced AAO template technique and Ag deposition. In a systematic study employing nanowires of various lengths, structures composed of aggregated AgNWs were optimized. The highest EF obtained for the aggregated AgNWs was ~2.3 × 10^7^, which was attributed to the large number of nanogaps between the Ag nanowires. From this experiment, the upper limit of EF for this nanostructure scheme was estimated to be <3 × 10^7^. When Ag was deposited on vertically aligned alumina nanowires before aggregation, an AgNP complex structure was formed instead of the AgNW structure. For the optimized AgNP complex structure, EFs as high as 3.5 × 10^7^ were obtained, of which ~2.5 × 10^7^ was estimated to be from the closely dispersed AgNPs on alumina nanowires in the slope region and ~10^7^ from the closely packed AgNPs in the roof region, as determined by micro-Raman measurements. The main mechanism for the large EF observed in the AgNP complex is not attributed to the hot spot effect but to an unknown physics of the AgNPs dispersed in 3D on the thin alumina nanowire framework. With this new AgNP scheme, novel nanostructures with EF > 10^8^ can be expected.

The novel Ag nanostructures used in this study are promising SERS media for highly sensitive chemical and biological sensors. For instance, they can be used to develop highly sensitive VOC gas sensors with sub-ppm- to ppb-level sensitivities. In these studies, the effective functionalization of molecular species to induce increased adsorption of target molecules on nanostructure surfaces is essential. Highly sensitive SERS films with EFs of >10^7^ will provide great competitiveness when accompanied by effective functionalized molecules. In particular, the AgNP complex substrate may be very effective for gas sensing because of its highly porous structure and excellent SERS activity. Macromolecules such as ZIF-8 are promising functional materials for this nanostructure to become capable of highly sensitive gas detection to protect life and property.

## Figures and Tables

**Table 1:** Nanostructure schemes proposed in this study and previously reported nanostructure schemes with enhancement factor (EF) > 10^7^, as selected based on the EF evaluation parameters.

Nanostructure Scheme	EF	Method *	Reference
Tilted AgNR array	7 × 10^8^	OAD	JPCC 2010 [7]
AgNW bundle array ^a^	1.4 × 10^8^	AAO; ED	Adv. Mat. 2015 [8]
AgNPs complex array	3.5 × 10^7^	AAO; TE	This work
Aligned AgNR array ^a^	3 × 10^7^	AAO; ED	JRS 2012 [15]
Aggregated AgNW’s array	2.3 × 10^7^	AAO; TE	This work
AgNP-coated Au nanofunnel array	2 × 10^7^	AAO; ED; TE	Sustainability 2022 [17]
Multilayered AgNP aggregation	1.5 × 10^7^	AgNP	Nanoscale 2011 [18]

* OAD: oblique angle deposition; AAO: anodic aluminum oxide; ED: electrodeposition; TE: thermal evaporation. ^a^ The nanostructure schemes actually belong to the same scheme.

## Data Availability

The data presented in this study are available on request from the corresponding author. The data are not publicly available due to privacy.

## Supplementary Materials

The following supporting information can be downloaded at: https://www.mdpi.com/article/10.3390/nano13243169/s1. Figure S1: Elemental mapping on Ag coated aggregated alumina nanowires of 5 μm by 60-nm thickness. Figure S2: Elemental mapping on Ag coated aggregated alumina nanowires of 5 μm by 100-nm thickness. Figure S3: Elemental mapping on AgNP complexes within aggregated alumina nanowires of 1 μm by Ag coating of 60 nm. Figure S4: Elemental mapping on AgNP complexes within aggregated alumina nanowires of 1 μm by Ag coating of 100 nm. Table S1: Quantitative elemental analysis results on Ag coated aggregated nanowires of 5 μm; Table S2: Quantitative elemental analysis results on AgNP complexes within aggregated alumina nanowires of 1 μm.
