# Supplementary material for: Ultrahighly Sensitive Surface-Enhanced Raman Spectroscopy Film of Silver Nanoparticles Dispersed in Three Dimensions on a Thin Alumina Nanowire Framework
Source: Nanomaterials (Basel). 2023 Dec 18;13(24):3169. doi: 10.3390/nano13243169 (PMC10745398; doi:10.3390/nano13243169)
Supplement: Supplementary file 1 [file nanomaterials-13-03169-s001.zip › nanomaterials-2726006-supplementary.pdf]

nanomaterials

MDPI

Supplementary Materials

Ultrahighly Sensitive Surface-Enhanced Raman Spectroscopy Film of Silver Nanoparticles Dispersed in Three Dimensions on a Thin Alumina Nanowire Framework

Myoung-Kyu Oh [1,*], Hyeonju Kim [1], Prince Gupta [2,3] and Kyoungsik Kim [3,*]

[1] Advanced Photonics Research Institute (APRI), Gwangju Institute of Science and Technology (GIST), Gwangju 61005, Republic of Korea; cpfl92811@gmail.com
[2] Carnot de Bourgogne Interdisciplinary Laboratory (Laboratoire Interdisciplinaire Carnot de Bourgogne), CNRS UMR 6303, University of Burgundy Franche-Comté (Université de Bourgogne Franche-Comté), 21000 Dijon, France; pri1989.iitr@gmail.com
[3] School of Mechanical Engineering, Yonsei University, Seoul 03722, Republic of Korea
* Correspondence: omkyu@gist.ac.kr (M.-K.O.); kks@yonsei.ac.kr (K.K.)

The morphological properties of the fabricated nanostructures, such as aggregated AgNW's and AgNP's complex structures were analyzed by a EDX spectrometer (Hitachi, S-4800; Horiba, EMAX). Qunatitative elemental analyses and Elemental mappings were performed on 2 aggregated AgNW's and 2 AgNP's complex samples as representitive samples, where aggretated alumina nanowires of 5 μm and vertically aligned alumina nanowires of 1 μm were deposited by 60 and 100 nm of Ag, respectively.

Table S1, S2 summarizes the quantitative elemental analysis results, where 250 x 250 μm^2 was selected as target area in each measurement.

Figure S1, S2 shows the elemental mapping results on Ag coated aggregated alumina nanowires of 5 μm by 60 and 100-nm thickness, respectively.

Figure S3, S4 shows the elemental mapping results on AgNP complexes within aggregated alumina nanowires of 1 μm by Ag coating of 60, 100 nm, respectively.

Table S1. Quantitative elemental analysis results on Ag coated aggregated nanowires of 5 μm. Ag coating thickness is denoted by the number in the sample names.

Elements	AgNW - 60 nm		AgNW - 100 nm	
	weight %	atomic %	weight %	atomic %
Ag	24.24	6.41	37.08	10.20
Al	57.29	60.62	35.63	39.18
O	18.47	32.96	27.29	50.62
Total	100.00		100.00	

Table S2. Quantitative elemental analysis results on AgNP's complexes within aggregated alumina nanowires of 1 μm. Ag coating thickness is denoted by the number in the sample names.

Elements	AgNP - 60 nm		AgNP - 100 nm	
	weight %	atomic %	weight %	atomic %
Ag	14.20	3.96	34.65	11.65
Al	85.30	95.10	64.85	87.21
O	0.50	0.94	0.50	1.13
Total	100.00		100.00	

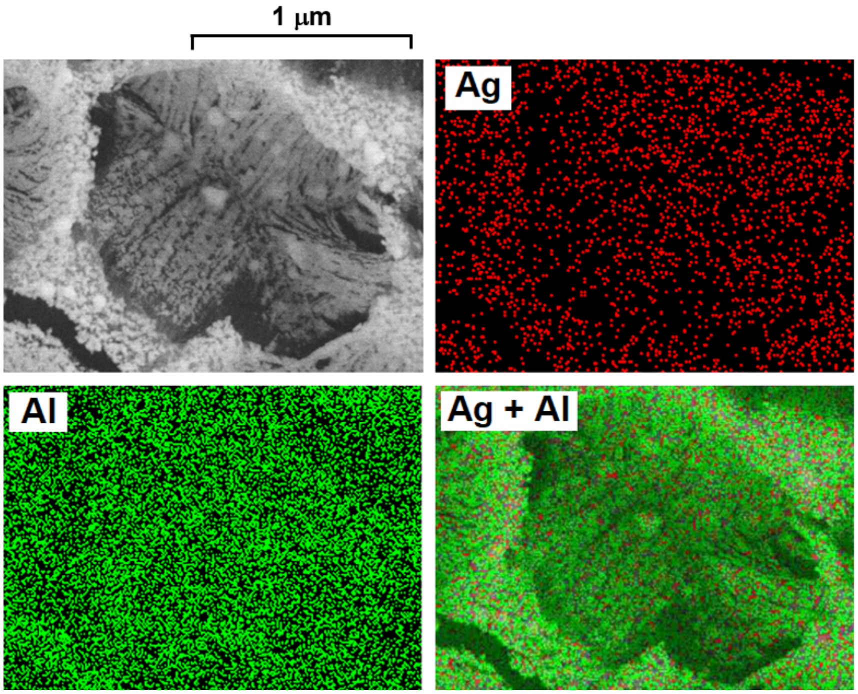

Figure S1. Elemental mapping on Ag coated aggregated alumina nanowires of 5 μm by 60-nm thickness.

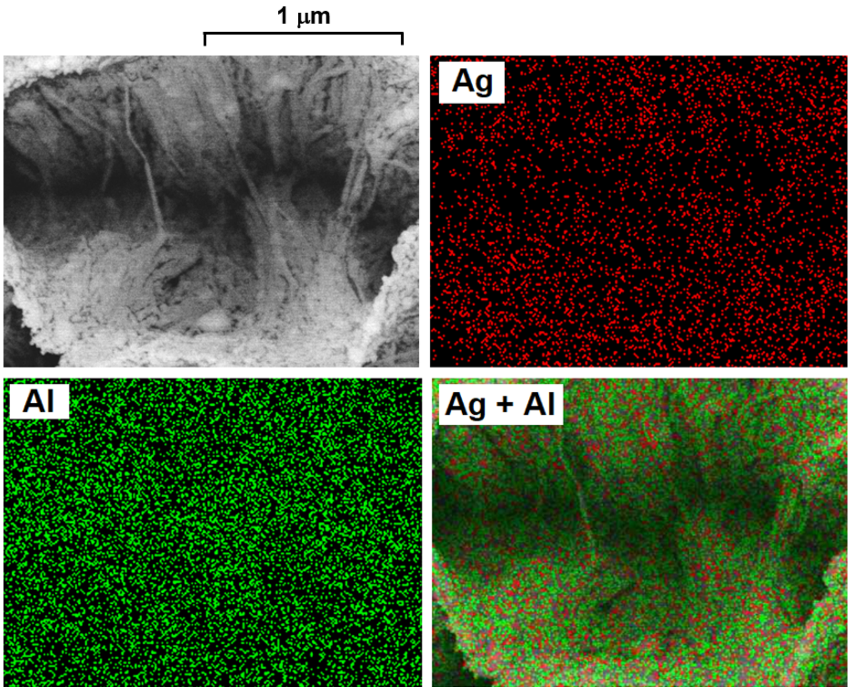

Figure S2. Elemental mapping on Ag coated aggregated alumina nanowires of 5 μm by 100-nm thickness.

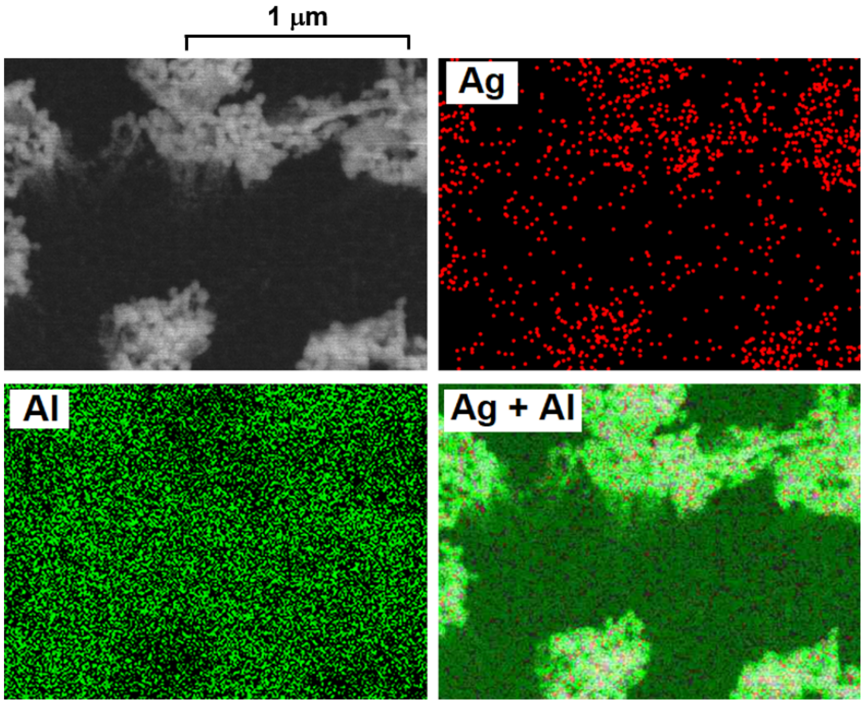

Figure S3. Elemental mapping on AgNP complexes within aggregated alumina nanowires of 1 μm by Ag coating of 60 nm.

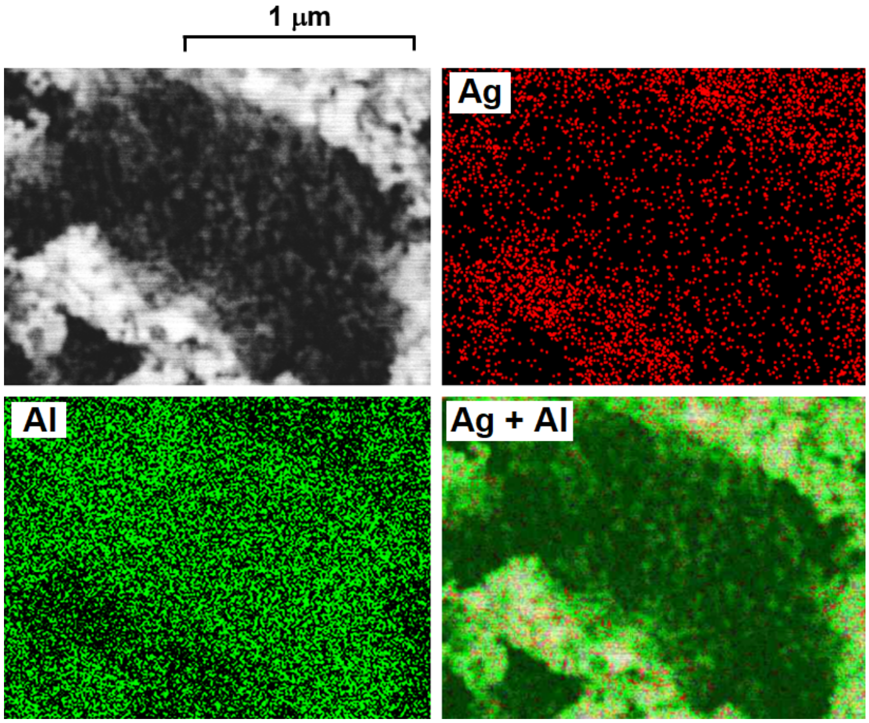

Figure S4. Elemental mapping on AgNP complexes within aggregated alumina nanowires of 1 μm by Ag coating of 100 nm.
